# How does asthma coexistence affect the strategic selection of biologic therapies in CRSwNP management?

**DOI:** 10.3389/falgy.2025.1579224

**Published:** 2025-03-31

**Authors:** Imran Ozdemir, Nuray Bayar Muluk, Mustafa Yazır, Cemal Cingi

**Affiliations:** ^1^ Department of Pulmonology, Medical Faculty, Üsküdar University, Istanbul, Türkiye; ^2^ Department of Otorhinolaryngology, Faculty of Medicine, Kırıkkale University, Kırıkkale, Türkiye; ^3^ Department of Otorhinolaryngology, Yazir Private Otorhinolaryngology Clinic, Izmir, Türkiye; ^4^ Department of Otorhinolaryngology, Faculty of Medicine, Eskisehir Osmangazi University, Eskisehir, Türkiye

**Keywords:** CRSwNP, asthma coexistence, biologic therapies, dupilumab, omalizumab, mepolizumab

## Abstract

**Objectives:**

We reviewed asthma coexistence and the selection of biologic therapies in CRSwNP Management.

**Methods:**

The literature review utilized Google and Google Scholar, in addition to PubMed, EBSCO, and Proquest Central at Kırıkkale University. We searched for “ CRSwNP”, “asthma”, “biologic therapies”, “Anti-IL-4RA”, “Dupilumab”, “Anti-IgE”, “Omalizumab”, “Anti-IL-5”, “mepolizumab” from 2024 to 2000.

**Results:**

Patients with CRSwNP frequently have co-occurring lower airway illnesses, including asthma and AERD asthma, which have a shared pathogenesis. The inflammatory bases of CRSwNP and asthma might be heterogeneous, with a type 2 or, less frequently, a non-type two inflammatory history. Lower airway inflammation and asthma control are worse in patients with asthma who also have CRSwNP. Patients with CRSwNP can now access targeted biologic medicines, a novel therapy option. The US Food and Drug Administration (FDA) has authorized three medications for CRSwNP: dupilumab, omalizumab, and mepolizumab. To treat chronic rhinosinusitis with a biological agent, the 2020 European position paper on rhinosinusitis established clear indications. A patient is considered a biologic therapy candidate if they have either undergone FESS before or did not meet FESS criteria but met three of the five. A diagnosis of concomitant asthma, necessitating an inhaled glucocorticoid controller regularly, is one of the five requirements.

**Conclusion:**

Biologic treatments have the potential to be used in certain patients where CRSwNP and asthma coexist. The recommended treatments include omalizumab, dupilumab, and mepolizumab.

## 1 Introduction

Asthma, chronic rhinosinusitis with nasal polyps (CRSwNP), and other lower airway illnesses share a common etiology and are frequently present in patients with CRSwNP. Asthma and CRSwNP are linked to various inflammatory factors, including type 2 and, less regularly, non-type two inflammatory backgrounds, whereas AERD is characterized by a single inflammatory factor: type 2. Severe, recurring disease and co-occurring involvement of the lower airways are common in patients with CRSwNP who mainly have a type 2 immunological profile. Medical and surgical treatments for CRSwNP with respiratory comorbidities (such as asthma and AERD) are more challenging, and patients experience worse quality of life (QoL), more severe sinonasal symptoms (such as nasal congestion and loss of smell), and longer treatment times. In typical clinical practice, the lungs and nose are handled independently even though the pathophysiological similarities between the two sets of airways have significant consequences for the diagnosis and treatment of these prevalent diseases. The case for targeted therapy that targets the immunological mechanisms of both diseases is strengthening as our knowledge of the pathophysiology of chronic upper and lower airway diseases grows (1).

## 2 Methods

Kırıkkale University's resources, including Google and Google Scholar, PubMed, EBSCO, and Proquest Central, were consulted for the literature study. Between the years 2024 and 2000, we looked for “CRSwNP”, “asthma”, “biologic therapies”, “Anti-IL-4RA”, “Dupilumab”, “Anti-IgE”, “Omalizumab”, “Anti-IL-5” and “mepolizumab”.

## 3 Comorbidity and severity of CRSwNP and asthma

Global Allergy and Asthma Network of Excellence (GA2LEN) data showed a high and persistent association between asthma and CRS (2). GA2LEN is a significant, multicenter, population-based epidemiological study. Estimates indicate that as many as 67% (range, 40%–67%) of patients with CRSwNP also have concomitant asthma, which is in line with the previously reported association between the two conditions (3, 4). Moreover, unrecognized asthma is common in many CRSwNP patients. According to Ragab et al. (5), most patients with CRSwNP had lower airway involvement, including 36% with minor airway illness and 24% with asthma. Among people with CRSwNP, the bronchial hyperresponsiveness test revealed 28%–40% had undetected asthma (6–9).

CRSwNP is typically not associated with asthma in children. Still, it is with adult late-onset (beginning after 40 years of age) or early-onset (beginning between 18 and 39 years of age) asthma in adults. In contrast, CRSsNP has been associated with two types of asthma in adults: childhood-onset (beginning before the age of 16) and early-onset (starting before the age of 40) (10, 11). It has been suggested that CRSwNP may be a risk factor for the severity of asthma, as it is more typically related to severe asthma (57.1%–62% of patients) than moderate asthma (38%–42.9% of patients) (12). The disease burden is expected to rise in an aging population because the prevalence of CRSwNP and accompanying asthma increases with age (13, 14).

When COPD and CRSwNP coexist, patients experience worse asthma control, more sputum eosinophilia, and worse lung function compared to those with COPD alone or with CRSsNP (1, 15).

## 4 The pathophysiology of CRSwNP

The pathophysiology of CRSwNP is characterized by a type 2 inflammatory signature, which may play a role in the development of NP, tissue remodeling, and the chronic inflammatory state of the sinonasal mucosa (16). While over 80% of instances in Caucasian populations are linked to a type 2 inflammatory signature in CRSwNP, Asian people with CRSsNP or CRSwNP are more likely to have an alternative inflammatory profile mediated by T helper (Th)1 and/or Th17 cells (17, 18). Key cytokines (IL-4, IL-5, and IL-13), type 2 chemokines (eosinophil cationic protein, eotaxin-1, eotaxin-2, eotaxin-3, pulmonary and activation-regulated chemokine, thymus and activation-regulated chemokine, and monocyte chemoattractant protein), and eosinophils show a significant increase in CRSwNP's type 2 inflammatory signature compared to healthy controls (16, 19, 20). Furthermore, NP biopsies from CRSwNP patients have also demonstrated increased innate lymphoid cells (ILC2), macrophages, and mast cells (18, 21–23).

In NP development, innate and adaptive immune system components interact with nasal mucosal remodeling, which includes changes in epithelial cells, epithelial-mesenchymal transition, goblet cell hyperplasia, degradation of the extracellular matrix, deposition of fibrin, and swelling of the tissues (21, 24). Patients with CRSwNP have NP with sinonasal epithelial barrier defects, such as decreased expression of tight junction and cell adhesion proteins (17, 21). Pathogens, proteases, and irritants can induce damage to the epithelium, which in turn triggers the release of cytokines that promote a Th2- immune response, including IL-25, IL-33, and thymic stromal lymphopoietin (TSLP) (25, 26). A type 2 inflammatory response can be amplified by the most highly triggered TSLP, which can activate type 2 ILC2s and mast cells and produce type 2 cytokines, including IL-5 and IL-13 (26, 27). According to *in vitro* findings, IL-4 and IL-13, type 2 cytokines, can promote TSLP production, perpetuating barrier dysfunction and the type 2 response (28).

Type 2 inflammation plays a crucial role in defending against parasitic infections and is a major factor in allergic diseases. This type of inflammation is driven by various immune cells, such as Th2 T cells, type 2 ILCs, eosinophils, mast cells, basophils, and IgE-producing B cells. The primary cytokines involved in type 2 responses are interleukin (IL)-4, IL-5, and IL-13. IL-4 and IL-13 contribute to the production of polyclonal IgE, with IL-4/IL-13 signaling promoting B cell class switching to IgE production (29, 30). Type 3 inflammation, on the other hand, is mainly responsible for defense against bacterial and fungal infections and is driven by Th17 cells and type 3 ILCs. The key cytokines in type 3 inflammation are IL-17 and IL-22 (29).

Multiple etiologies (such as epithelial barrier dysfunction, microbiome imbalance, and mucociliary dysfunction) lead to immunological dysfunction and chronic inflammation in CRSwNP patients, impairing the host-environment interaction at the sinonasal mucosa. In addition to worsening inflammation, pathogens can trigger innate and adaptive immune responses when the epithelial barrier is dysfunctional (31). Staphylococcus aureus, which was found in 63% of CRSwNP patients in a prior investigation, secretes enterotoxins that can stimulate the development of IgE antibodies that are specific to antigens, leading to an increase in type 2 inflammatory responses (1, 18).

Additional pathophysiological studies have revealed various inflammatory patterns, which gave rise to the concept of “endotyping of CRS”. Endotyping primarily aims to identify the predominant inflammatory type to guide more targeted treatment strategies. The current framework suggests distinguishing between type 2 (eosinophilic) and non-type 2 inflammatory responses (32).

## 5 Choosing between surgery and biologıc therapy in CRSwNP

For CRS patients never operated before, we usually recommend they consult an otolaryngologist about functional endoscopic sinus surgery (FESS) before starting biologic treatment. A cohort analysis evaluated the effectiveness of FESS with three biologic therapies—dupilumab, omalizumab, and mepolizumab—as reported in randomized trials comparing each biologic to placebo (33, 34). This preference is based on the results of those trials. Due to a substantial decline in FESS patients during follow-up, this study may have been biased. Conflicts over participant autonomy and blinding have prevented direct comparisons of surgical procedures with biological treatments (33).

With failure of initial medical care 111 CRSwNP patients underwent FESS and were assessed at 24 and 52 weeks in a multicenter cohort trial (34). At 24 weeks Sino-Nasal Oucomes Test (SNOT-22) scores were more improved by FESS as compared to dupilumab (in one trial) and omalizumab (in two trials). At 52 weeks the SNOT-22 scores were similar for dupilumab end FESS. FESS also showed better efficacy compared to mepolizumab. As anticipated, FESS significantly reduced polyp load compared to the biologics at both periods. Because biologics, in theory, need to be continued permanently, FESS seems to be a more cost-effective alternative to biologic therapy (35–38).

While it is generally recommended that patients with CRSwNP undergo FESS before beginning biologic therapy, there are notable exceptions (33):

– Individuals whose asthma is so poorly managed that biologic therapy is necessary for the treatment of their condition, regardless of whether or not they experience sinus problems

People who who refuse or can not go to the surgery for any other contra indications of the surgery following a joint decision-making process.

## 6 Biologic therapies

One recent development in treating CRSwNP is biological treatments (39–43). The US Food and Drug Administration (FDA) has authorized three medications for CRSwNP: dupilumab, omalizumab, and mepolizumab.

Currently, researchers are focusing on the practical and long-term use of biologics in CRSwNP (33).

### 6.1 Indications

When patients who have had functional endoscopic sinus surgery (FESS) experience a disease recurrence after the procedure, most CRS guidelines recommend respiratory biologic therapy (34, 36–38).

Specific indications for biologic therapy for CRSwNP were proposed in the 2020 European position document on rhinosinusitis (44). Extra changes were made to them in 2023 (45). Patients who were considered biologic candidates either had prior FESS experience or did not meet FESS eligibility requirements but did meet three out of five (33, 44):

– “Evidence of type 2 inflammation (sinus tissue eosinophil count ≥10 eosinophils per high-powered field or peripheral blood eosinophils ≥150 cells/ul or total IgE ≥100 international units/ml)”
– “Need for oral glucocorticoids ≥2 courses per year or ≥3 months of low-dose oral glucocorticoids or a contraindication for systemic glucocorticoids”
– “Significant impairment in quality of life [Sino-Nasal Outcomes Test (SNOT-22) score ≥40]”
– “Significant loss of smell (anosmia on objective smell testing)”
– “Diagnosis of comorbid asthma (requiring at least a regular controller inhaled glucocorticoid)”

### 6.2 Pretreatment evaluation

It is common practice to assess markers of type 2 inflammation, such as peripheral blood eosinophil counts and serum IgE levels, before beginning biologic therapy. Elevations in these parameters may lend credence to the biological decision. Evaluating these biomarkers might be more challenging once a patient has started treatment since medication can alter them. The alternative diagnosis of eosinophilic granulomatosis with polyangiitis (EGPA) should be considered in patients with peripheral blood eosinophilia, nasal polyps, and asthma. This is because biological treatment can potentially alleviate pulmonary and nasal symptoms while hiding the underlying vasculitis (33).

The presence of Eosinophilic-rich mucus (ERM) in sinonasal secretions during surgery appears to be a predictor for recurrence and the potential need for follow-up surgery (46).

### 6.3 Role of endotyping

Endotypes are a way to categorize CRS according to its pathophysiology and underlying inflammation profiles found by gene expression and biomarkers (47). A phenotype, on the other hand, is a set of observable clinical traits. Some therapeutic options for CRSwNP can be informed by phenotype-based classifications, such as NSAID hypersensitivity in patients with aspirin-exacerbated respiratory disease [AERD]. Patients with CRSwNP may not always respond well to respiratory biologics that target type 2 inflammation, and phenotypic differences may not necessarily indicate the underlying inflammatory mechanisms that cause CRSwNP (48, 49).

Type 2 innate lymphoid cells (ILC2s), tissue eosinophilia, type 2 cytokines, mast cell infiltrates, and local IgE generation are the hallmarks of CRSwNP in Western Europe and the United States (50, 51). Conversely, certain patients exhibit tissue-level inflammatory endotypes such as type 1 (IL-12 driven), type 3 (IL-17 driven), or a combination of the two (51–53).

Patients with signs of type 2 inflammation, such as elevated levels of tissue and peripheral blood eosinophils (polyp tissue eosinophils ≥10/high-power field or blood eosinophils ≥150 cells/mcL) and serum IgE (total IgE ≥100 international units/ml), should be considered for biologic therapy treatment of CRSwNP, according to current guidelines (44, 45). Nevertheless, despite differences in baseline eosinophil level, aspirin sensitivity, concomitant asthma (54), and concomitant allergic rhinitis, subgroup evaluations of biologic treatment for CRSwNP management have demonstrated that therapy is effective. Therefore, more research is required to identify patient-specific factors that indicate which medicine will have the most significant impact.

In Vlaminck et al.'s study, among the 133 patients, 81% exhibited local eosinophilia, and 60% had eosinophilic-rich mucus (ERM). Recurrence occurred in 62% of cases during follow-up and was linked to both local eosinophilia and ERM (both *p* < 0.001). Additionally, patients who experienced recurrence were more likely to have ERM and fungal hyphae (FH), with statistical significance for ERM (*p* < 0.001), FH (*p* < 0.001), and fungal hyphae (*p* = 0.008) (46).

### 6.4 Selecting among biologic agents

The US and EU have authorized using three biologics classified as “respiratory” (i.e., appropriate for treating asthma): dupilumab, omalizumab, and mepolizumab. No randomized controlled trials comparing these drugs have been conducted to treat CRSwNP. Nevertheless, several systematic evaluations and indirect comparisons consistently indicated that dupilumab was the most effective (55–58).

All three medications have received the green light for moderate to severe asthma. Patient comorbidities and particular laboratory findings can also play a role in guiding biologic selection; for example, in cases where a patient has two indications for biologic therapy—for example, atopic dermatitis, eosinophilic esophagitis, or chronic urticaria—the secondary indication can guide the choice of biologic. Endotyping as a tool for respiratory biologic drug selection is still in its early stages and needs more research (59, 60).

Researchers evaluated the effects of biologic treatment on health-related quality of life (SNOT-22 score), disease severity, and significant adverse events in a meta-analysis of ten randomized trials of dupilumab, omalizumab, or mepolizumab for CRS (with nearly all participants having CRSwNP). When given dupilumab, omalizumab, and mepolizumab, SNOT-22 scores increased by 19.6 points, 15.6 points, and 13.3 points, respectively (58). In terms of the SNOT-22 score, all three agents were able to reach the MCID of 8.9 points (33).

According to an indirect treatment comparison study of CRSwNP (53), dupilumab was better than omalizumab, except that the groups’ SNOT-22 scores were comparable. An ongoing research compares omalizumab and dupilumab as treatments for CRSwNP (NCT04998604) (33).

Dupilumab showed superior results compared to omalizumab and mepolizumab in improving quality of life, as indicated by the SNOT-22 score, nasal obstruction, smell improvement, reduced reliance on rescue oral corticosteroids, and a lower need for ESS. It also outperformed the others in reducing nasal polyp size, enhancing endoscopic appearance (Lund-Kennedy endoscopy score), and improving CT scores (Lund-Mackay CT score) (61).

However, in patients with moderate-to-severe serum eosinophilia, dupilumab might not be recommended, as it could increase peripheral eosinophilia and potentially trigger or worsen EGPA. In such cases, mepolizumab might be a more suitable option. Furthermore, mepolizumab is FDA-approved for treating EGPA (62).

When added to standard treatment, benralizumab reduced the nasal polyp score (NPS), alleviated nasal congestion, and improved the sense of smell in patients with CRSwNP compared to a placebo (63, 64). Tezepelumab, a human monoclonal antibody that inhibits thymic stromal lymphopoietin from binding to its receptor, decreased asthma exacerbations and reduced type 2 inflammatory biomarkers in patients with and without nasal polyps (65–67).

A *post hoc* analysis of the DREAM and QUEST studies suggests that a combination of baseline blood eosinophil count and FeNO levels might help predict responses to treatment with mepolizumab and dupilumab (68, 69). However, the role of these markers combined with periostin in predicting treatment outcomes is less well-defined (70, 71).

#### 6.4.1 Anti-IL-4RA (dupilumab)

An essential factor in disorders like CRSwNP and asthma is type 2 inflammation, which is dupilumab, a monoclonal antibody that targets interleukin four receptor alpha (IL-4R-alpha), blocks. When administered orally, dupilumab improves smell, decreases nasal congestion/blockage, inflammation of the endoscopic and radiologic sinuses, rhinorrhea, postnasal drip, the requirement for oral glucocorticoids, and the necessity for FESS in patients with chronic rhinorrhea syndrome with nasal polyps (31, 72). Patients with AERD exhibit nearly uniform improvement with dupilumab (73, 74), suggesting that it may be especially effective in this population. Therefore, while treating CRSwNP with biologic therapy, we usually pick dupilumab for patients with AERD. Additionally, it has the green light for treating atopic dermatitis, prurigo nodularis, eosinophilic esophagitis, and asthma (33).

#### 6.4.2 Anti-IgE (omalizumab)

Nasal polyp tissue contains local immunoglobulin E (IgE), and patients with more severe CRSwNP have higher levels of IgE, which can be addressed with the anti-IgE medication Omalizumab (50, 75). Patients with CRSwNP taking omalizumab report less nasal polyp burden and sinonasal symptoms, according to older observational studies and newer randomized, placebo-controlled trials (76–81). Allergic asthma and chronic spontaneous urticaria are two more conditions that omalizumab effectively treats (33).

#### 6.4.3 Anti-IL-5 (mepolizumab)

Mepolizumab targets interleukin (IL) 5, which is essential for the survival and activation of eosinophils. It has been found that nasal polyp tissue has an increased number of eosinophils, contributing to the inflammation that causes CRSwNP (82). Patients with CRSwNP who use mepolizumab instead of a placebo report less nasal congestion, higher quality of life, and a higher NPS (48). It also treats eosinophilic asthma, hypereosinophilic syndrome, and eosinophilic granulomatosis with polyangiitis (EGPA) (33).

### 6.5 Conclusion

Currenty there is an indication for the use of biologic therapies either in patients with recurrence after FESS or in those not eligible for FESS for different reasons when three out of five criteria have been met (33, 44). Short term and long term success cannot be predicted at this day for the lack of clear paremeters in predicting outcomes.
